# CD44 targeting reduces tumour growth and prevents post-chemotherapy relapse of human breast cancers xenografts

**DOI:** 10.1038/sj.bjc.6604953

**Published:** 2009-02-24

**Authors:** E Marangoni, N Lecomte, L Durand, G de Pinieux, D Decaudin, C Chomienne, F Smadja-Joffe, M-F Poupon

**Affiliations:** 1 Translational Research Department, Institut Curie, 75010 Paris, France; 2 INSERM, UMR-S-718, Institut Universitaire d’Hématologie, Hôpital St-Louis, 16 rue de la Grange aux belles, 75010, Paris, France; 3 Department of Pathology, Université François Rabelais Hôpital Trousseau, Tours, France

**Keywords:** breast cancer, CD44, xenografts

## Abstract

CD44 is a marker of tumour-initiating cells and is upregulated in invasive breast carcinoma; however, its role in the cancer progression is unknown. Here, we show that antibody-mediated CD44-targeting in human breast cancer xenografts (HBCx) significantly reduces tumour growth and that this effect is associated to induction of growth-inhibiting factors. Moreover, treatment with this antibody prevents tumour relapse after chemotherapy-induced remission in a basal-like HBCx.

Breast cancer remains a leading cause of morbidity and mortality in women. The long-term survival of patients with advanced breast cancers is still poor despite recent therapeutic improvements (Kamangar *et al*, 2006). The main reasons for the poor clinical outcomes are the local and distant tumour recurrences. New targets are therefore needed to improve successful treatments. The CD44 glycoprotein is highly expressed in breast tumours and this overexpression has been described as a poor prognostic factor (Gotte and Yip, 2006). CD44 is involved in several processes, including tumour cell proliferation, adhesion and invasion (Ponta *et al*, 2003). Interestingly, CD44 was also found to be a positive marker of tumour-initiating cells in different types of tumours, including breast cancers (Al-Hajj *et al*, 2003). *In vivo* CD44 targeting by siRNA and by specific antibody results in anti-tumor activity in colon cancer xenografts and human acute leukaemia xenografts, respectively (Jin *et al*, 2006; Subramaniam *et al*, 2007). In breast cancer, the role of CD44 in cell proliferation is still unclear. To determine if CD44 targeting could affect tumour growth *in vivo,* we used the P245 monoclonal antibody against the CD44 cell surface receptor that was earlier shown to inhibit the development of human leukaemia xenografts (Jin *et al*, 2006).

## Materials and methods

### Mice, compounds, treatment and tumour growth measurement

Female Swiss nude mice, 10-week old, were purchased from Charles River (Les Arbresles, France) and maintained in specific pathogen-free conditions. Their care and housing were in accordance with institutional guidelines as put forth by the French Ethical Committee (Agreement B75-05–18, France) under the supervision of an authorised investigator (MF Poupon).

The murine anti-CD44 monoclonal antibody (P245) and Hermes3 were supplied by Laurence Boumsell (INSERM, Paris, France) and David Naor, respectively, and the isotype control (IgG1) was from Sigma-Aldrich (St Louis, MO, USA). Adriamycin (Doxorubicin) and cyclophosphamide (Endoxan) were purchased from Teva Pharmaceuticals (Paris, France) and Baxter (Maurepas, France), respectively. Adriamycin and cyclophosphamide combination was designated as AC. Hyaluronan (HA, MW: 5 × 10^4^ to 2 × 10^6^ Da) was provided by Javenech Cie (Fougères, France).

Human breast cancer xenografts, HBCx-3, -8 and -10, were established as detailed (Marangoni *et al*, 2007). Treatments were done on groups of eight animals (HBCx-3 and HBCx-8) and 12 animals for the tumour recurrence experiment (HBCx-10).

### Immunohistological staining and flow cytometry analysis

CD44^+^ cells were searched for by immunochemistry in formol-fixed tumour histological sections, prepared as described earlier (Marangoni *et al*, 2007) and stained with biotin-conjugated anti-CD44 mAb Hermes-3 (Picker and Butcher, 1992). The frequency of CD44^+^ cells was assessed in tumours by flow cytometry. A single-cell suspension was prepared from tumours by gentle mechanical crushing, and filtration through cotton gauzes and enzymatic digestion (0.05% trypsin/0.025% EDTA). After washing, cells were labelled with FITC-conjugated P245 anti-CD44 mAb (1 μg per 10^5^ cells) or IgG1 (50-fold diluted, BD Biosciences, Le Pont-De-Claix, France). The proportion of CD44^+^ cells was measured by flow cytometry relative to isotype-matched controls, using a FACSvantage (Becton Dickinson, San Jose, CA, USA). Each measurement was done on 5,000 cells. Dead cells were excluded using 7-AAD.

### RT–PCR analysis

CD44 mRNA expression was analysed by nested RT–PCR using human specific intron-spanning primers (designed with the PrimerExpress Software, Applied Biosystems, Courtaboeuf, France) from mRNA of tumours or fat pad tissues frozen in liquid nitrogen and extracted as described earlier (Delaunay *et al*, 2007). The mRNA levels of proinflammatory human cytokines (IL-1b, TGFb1, Oncostatin M and TNF-α) were measured by real-time quantitative RT–PCR, using TaqMan Gene Expression Assays with Assays-on-Demand (Applied Biosystems), and carried out with TaqMan universal PCR master mix (Applied Biosystems), and with an ABI PRISM 7700 Sequence Detection System (Applied Biosystems). mRNA expression of the human porphobilinogen deaminase gene was used as an internal standard.

The differences between median Δ*C*_T_ values from P245-treated and control groups were considered as statistically significant for *P*⩽0.05 (unpaired Student's *t* test).

## Results

**Figure 1:**
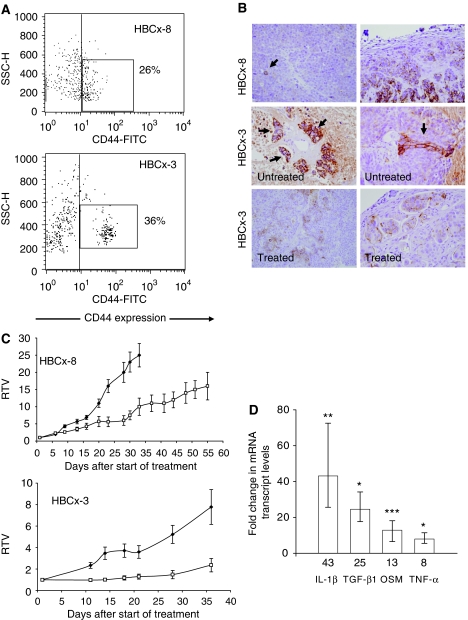
CD44 expression in human breast cancer xenografts (HBCx) and effects of P245 anti-human CD44 mAb on HBCx growth. (**A**) Flow cytometry assay of CD44^+^ cells in HBCx-8 and HBCx-3 xenografts. (**B**) Immunohistology of HBCx stained with biotin-conjugated Hermes-3 anti-human specific CD44 antibody. Haematoxylin/Eosin sections × 200. Upper: Untreated HBCx-8; a single strongly positive CD44^+^ cancer cell (arrow) isolated among CD44^−^ cancer cells (left): membrane staining is seen, with a heterogeneous pattern. Untreated HBCx-8 tumour; a gradient of staining is observed from the periphery to the central areas (right). Untreated HBCx-3 tumour; strong staining of surviving clumps of cells (arrows), within necrotic areas in the centre of the tumour. Middle: Untreated HBCx-3 showing low staining of CD44^+^ cells in the periphery of the tumour. Bottom: HBCx-3 after treatment showing residual CD44^+^ cells. (**C**) Anti-tumoural effects of P245 mAb on tumour growth. Growth curves; relative tumour volume (RTV) of HBCx-8 (upper) and HBCx-3 (bottom) as a function of time (days after start of treatment), IgG1 isotype control (black squares) or P245-treated (white squares) with P245 mAb. Mice bearing HBCx-3 and HBCx-8 tumours, at a mean volume of 100 mm^3^, were randomized into treatment and control groups (8 to 12 mice per group). The P245 mAb was injected intraperitoneally (i.p.) at a dose of 3 mg kg^−1^ in 100 *μ*l of 0.9% NaCl, thrice weekly for 4 to 8 weeks. The control group was not treated or received IgG1injections (the same dose and protocol as for P245 mAb). The RTV for each mouse was the ratio of tumour volume at time *t* to the initial volume. Mean RTV and standard errors were calculated. Mice were ethically killed when the tumour volume reached 2500 mm^3^. Statistical significance of observed differences was calculated by the paired Student′s *t* test by comparing the RTV in the treated (*T*) and control (*C*) groups for the tumour growth curves and by the *χ*^2^ test for the tumour relapse frequencies. (**D**) Embedded gene expression changes induced by anti-CD44 mAb treatment of HBCx-8: mRNA levels of proinflammatory human cytokines (IL-1β, TGFβ1, Oncostatin M and TNF-7α) were measured by real-time quantitative RT–PCR. mRNA was extracted from frozen tumours excised at the end of P245 treatment. Shown are results from one representative of three experiments. Each sample was run in duplicate and the mean value was expressed as cycle threshold (CT), ^*^⩽0.05, ^**^⩽0.02, ^***^⩽0.05.

Two different HBCx, originating from aggressive primary tumours (Marangoni *et al*, 2007), were used to test the effect of P245 mAb on tumour growth. CD44 expression was analysed by both FACS analysis and immunohistochemistry (IHC). FACS analysis indicated that HBCx-8 and HBCx-3 consisted of 25% and 36% CD44^+^ cells (Figure 1A), respectively, with a heterogeneous staining in both xenografts showed by IHC (Figure 1B). In HBCx-8 few isolated strongly staining cells (Figure 1A) were observed in the well-vascularised tumour periphery with a progressive increase in CD44^+^ cells towards the hypoxic/necrotic central area (Figure 1A). In HBCx-3 a very strong CD44 staining was found in cancer cell islets near within the necrotic areas (Figure 1A).

To evaluate the anti-tumoral effects of CD44 targeting *in vivo*, the P245 mAb was injected intraperitoneally three times weekly into nude mice. The tumour growth inhibition (TGI) in the P245 mAb-treated group was 70% at day 28 (*P*<0.01) in both xenografts, with HBCx-3 tumour growth being fully stabilized as early as day 5, whereas HBCx-8 was strongly inhibited from days 15–20 (Figure 1C). After cessation of the antibody treatment (at day 51), HBCx-8 growth delay was prolonged 2.5-fold when compared with the control group receiving murine irrelevant IgG_1_.

At the end of treatment, tumours were excised and analysed for proliferation and apoptotic markers and cytokines induction known to be associated to CD44 receptor ligation by specific antibodies (Delaunay *et al*, 2007). Whereas no changes were found in Ki67 or apoptosis markers (data not shown), P245 mAb treatment was associated with increased expression of genes encoding for human IL-1β, TGF-β1, OSM and TNF-α by 43-, 24-, 13- and 8-fold, respectively (Figure 1D).

**Figure 2:**
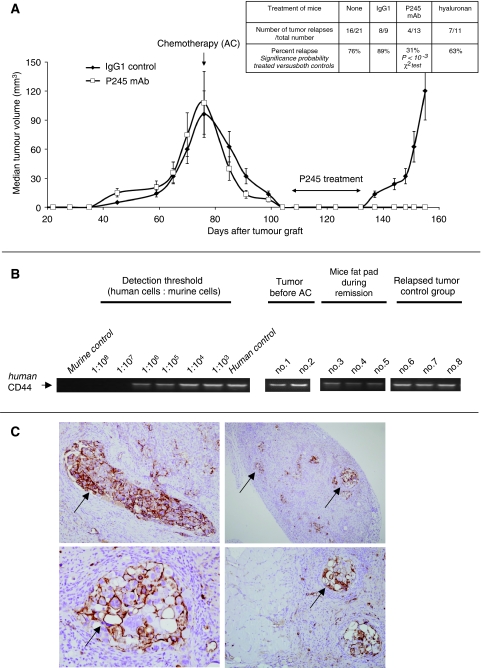
HBCx-10 recurrences after adriamycin/cyclophosphamide (AC) chemotherapy and their prevention by P245 mAb treatment. Detection of human CD44 transcripts in the post-chemotherapy tumour residue. (**A**) Tumour growth curves of HBCx-10 after chemotherapy alone or chemotherapy followed by P245 anti-CD44 mAb treatment. Tumour bearing mice were all treated with the AC combination (adriamycin 2 mg kg^−1^, cyclophosphamide 100 mg kg^−1^) when the tumour reached a median volume of 100 mm^3^. Mice were then separated into two groups until tumour remission was complete (i.e., the tumours were not palpable). The y axis shows the tumour volume median. One group was treated three times per week with P245 anti-CD44 mAb (3 mg kg^−1^ for 5 weeks). The control group was untreated. (**B**) Detection of human CD44 transcripts in the residual fat pad tissue during the post-chemotherapy remission. CD44 mRNA expression was analysed by nested RT–PCR using human specific intron-spanning primers (designed with the PrimerExpress Software, Applied Biosystems) from mRNA of tumours or fat pad tissues frozen in liquid nitrogen and extracted as described earlier. Cross-species amplification and detection threshold of human CD44-transcribing cells was determined with a concentration range of human breast cancer cells (MDA-MB231) in murine fibroblasts (NIH-3T3). Data are shown as the human CD44 signal obtained after two amplification sets of 2 μg of cDNA from HBCx-10 tumours before adriamycin/cyclophosphamide (AC) treatment (mice no.1 and 2), during the relapse in the control group (mice no. 6, 7 and 8) compared with mice fat pad during complete remission (mice no. 3, 4 and 5). Shown are results from one representative of three experiments. (**C**) Immunohistology of HBCx-10 tumour nodules during tumour remission stained with biotin-conjugated Hermes-3 anti-human specific CD44 antibody. Haematoxylin/Eosin sections × 400 (left) and × 200 (right). Left: single tumour cell nodules strongly positive for CD44 (arrow) isolated among unstained small murine cells. Right: murine tissue containing adipocytes and stroma with disseminated clumps of human tumour cells expressing CD44 (arrows).

We next addressed the role of CD44^+^ cells in initiating tumour recurrences after conventional therapy. We took advantage of the triple-negative HBCx-10, which is exquisitely sensitive to combined adriamycin/cyclophosphamide (AC), a treatment commonly used for this breast cancer subtype (Rouzier *et al*, 2005). A single AC injection in mice with a medium tumour volume of 100 mm^3^ (usually 75 days after tumour graft) induces a complete regression of all tumours by day 35, both by local palpation and histological analysis of the injected fat pads Figure 2A. At this period of complete remission, human CD44 transcripts are detected by semiquantitative nested RT–PCR, in the fat pads at the site of the tumour graft (detection threshold huCD44^+^ cells: one in every 10^7^ cells) (Figure 2B), showing that at least a fraction of CD44^+^ cells were resistant to AC. The presence of CD44^+^ cells during tumour remission is confirmed by IHC analysis (Figure 2C). Small islets of human tumour cells strongly stained for CD44 are visible in the mouse tissue fat pad. In fact, HBCx-10 tumours recurred locally with high frequency, affecting 16 of 21 mice (76%) after 4–6 weeks of complete tumour regression (see Figure 2A). To test whether CD44 targeting could affect tumour relapse, the P245 mAb was injected during the tumour remission. As shown in Figure 2A, treatment by the P245 mAb decreased the frequency of tumour recurrence to 31% (31%, *P*<10^−3^), whereas injections of IgG1 isotype control had no effect. These results support the idea that CD44^+^ surviving cells play a role in tumour recurrences.

## Discussion

Here we show for the first time that CD44 targeting with the P245 mAb strongly inhibits the growth of HBCx, suggesting that CD44^+^ cells play an important role in tumour growth-driving mechanisms. This effect was associated with the activation of cytokine gene expression, suggesting that these cytokines may be involved in the P245-mediated inhibition of tumour growth (Pagliacci *et al*, 1993; Derynck *et al*, 2001; Shen *et al*, 2002; Underhill-Day and Heath, 2006). Interestingly, inhibition of breast cancer stem cells by TGF-β1 was reported recently (Tang *et al*, 2007) and TGF-β1 signalling is functional in CD44^+^ breast cancer cells (Derynck *et al*, 2001). In the treated tumours, there was no evidence of decreased expression of the cell proliferation markers such as Ki67 or activation of apoptotic pathways. Moreover, the fact that tumours regrew on stopping the treatment suggests that the P245 mAb did not completely eradicate all malignant cells. In fact, CD44^+^ cells still persist in the treated HBCx-3 at the end of treatment, as shown by IHC, suggesting that CD44 targeting could have a cytostatic effect. To improve the therapeutic benefit of CD44 targeting, it might be necessary either to increase CD44 accessibility, or P245 dosage or length of administration. CD44-targeted therapy of breast cancer might therefore require a continuous administration of the P245 mAb, as is the case for other antibody-based therapies, such as with the anti-HER2 mAb (trastuzumab) (Hudis, 2007). As HA binds to CD44 receptor, we asked whether HA could also inhibit the post-chemotherapy recurrence of breast cancer xenografts. We found that administration of exogenous HA, at the same dose and the same frequencies as P245, did not decrease significantly the frequency of tumour recurrence (Figure 2A). This result is in agreement with the otherwise published results reporting that HA usually does not inhibit tumour growth. Interestingly, P245 mAb does not block HA binding to mammary tumour cells (data not shown), indicating that P245 and HA bind distinct regions of the CD44 protein. Therefore, it is not surprising that HA does not recapitulate the TGI provoked by P245, as it is now well established that the engagement of distinct regions of CD44, with specific mAbs or with HA, triggers distinct biological effect (Müller-Sieburg *et al*, 2000; Gadhoum *et al*, 2004).

In breast cancer treatment, combined chemotherapy and radiotherapy can induce disease remission; however, tumour recurrences are frequent and responsible for treatment failures. Such recurrences are due to the persistence and proliferation of chemoresistant breast cancer cells. This is particularly true for basal-like breast cancers, which are sensitive to anthracyclines and taxanes combinations, but have higher risk of recurrence after therapy and worse survival rates (Fadare and Tavassoli, 2008). By using a tumour xenograft originating from a human basal-like breast cancer, we show that the eradication of the large bulk of tumour cells by chemotherapy is associated with the presence of CD44^+^ cancer cells, suggesting that a subpopulation of CD44^+^ tumour cells survives in spite of the macroscopic complete tumour remission induced by chemotherapy. The fact that P245 treatment during tumour remission inhibits tumour recurrence suggests that CD44^+^ cells play a direct role in the tumour recurrence, providing an interesting target and a useful model to finalise breast cancer therapies associations.

In conclusion, we show here for the first time that targeting CD44^+^ breast cancer cells inhibits tumour growth and post-chemotherapy tumour recurrence in both ER+ and basal-like breast cancer. In addition, we show that the effect of P245 mAb was associated with induction of cytokines known to have anti-proliferative activity. The finding that CD44^+^ cells play a key role in chemoresistant breast cancer recurrences provides an innovative strategy to improve breast cancer treatment.

## References

[bib1] Al-Hajj M, Wicha MS, Benito-Hernandez A, Morrison SJ, Clarke MF (2003) Prospective identification of tumorigenic breast cancer cells. Proc Natl Acad Sci USA 100: 3983–3988 12629218 10.1073/pnas.0530291100 PMC153034

[bib2] Delaunay J, Lecomte N, Bourcier S, Qi J, Gadhoum Z, Durand L, Chomienne C, Robert-Lezenes J, Smadja-Joffe F (2007) Contribution of GM-CSF and IL-8 to the CD44-induced differentiation of acute monoblastic leukemia. Leukemia 22(4): 873–876 17914409 10.1038/sj.leu.2404976

[bib3] Derynck R, Akhurst RJ, Balmain A (2001) TGF-beta signaling in tumor suppression and cancer progression. Nat Genet 29: 117–129 11586292 10.1038/ng1001-117

[bib4] Fadare O, Tavassoli FA (2008) Clinical and pathologic aspects of basal-like breast cancers. Nat Clin Pract Oncol 5: 149–159 18212769 10.1038/ncponc1038

[bib5] Gadhoum Z, Delaunay J, Maquarre E, Durand L, Lancereaux V, Qi J, Robert-Lezenes J, Chromienne C, Smadja-Joffe F (2004) The effect of anti-CD44 monoclonal antibodies on differentiation and proliferation of human acute myeloid leukemia cells. Leuk Lymphoma 45(8): 1501–1510 15370200 10.1080/1042819042000206687

[bib6] Gotte M, Yip GW (2006) Heparanase, hyaluronan, and CD44 in cancers: a breast carcinoma perspective. Cancer Res 66: 10233–10237 17079438 10.1158/0008-5472.CAN-06-1464

[bib7] Hudis CA (2007) Trastuzumab—mechanism of action and use in clinical practice. N Engl J Med 357: 39–51 17611206 10.1056/NEJMra043186

[bib8] Jin L, Hope KJ, Zhai Q, Smadja-Joffe F, Dick JE (2006) Targeting of CD44 eradicates human acute myeloid leukemic stem cells. Nat Med 12: 1167–1174 16998484 10.1038/nm1483

[bib9] Kamangar F, Dores GM, Anderson WF (2006) Patterns of cancer incidence, mortality, and prevalence across five continents: defining priorities to reduce cancer disparities in different geographic regions of the world. J Clin Oncol 24: 2137–2150 16682732 10.1200/JCO.2005.05.2308

[bib10] Marangoni E, Vincent-Salomon A, Auger N, Degeorges A, Assayag F, de Cremoux P, de Plater L, Guyader C, De Pinieux G, Judde JG, Rebucci M, Tran-Perennou C, Sastre-Garau X, Sigal-Zafrani B, Delattre O, Dieras V, Poupon MF (2007) A new model of patient tumor-derived breast cancer xenografts for preclinical assays. Clin Cancer Res 13: 3989–3998 17606733 10.1158/1078-0432.CCR-07-0078

[bib11] Müller-Sieburg CE, Deryugina E, Khaldoyanidi S, O'Rourke A (2000) Tissue- and epitope-specific mechanisms account for the diverse effects of anti-CD44 antibodies on the maintenance of primitive hematopoietic progenitors *in vitro*. Blood Cells Mol Dis 26(4): 291–302 11042030 10.1006/bcmd.2000.0306

[bib12] Pagliacci MC, Fumi G, Migliorati G, Grignani F, Riccardi C, Nicoletti I (1993) Cytostatic and cytotoxic effects of tumor necrosis factor alpha on MCF-7 human breast tumor cells are differently inhibited by glucocorticoid hormones. Lymphokine Cytokine Res 12: 439–447 8123760

[bib13] Picker LJ, Butcher EC (1992) Physiological and molecular mechanisms of lymphocyte homing. Annu Rev Immunol 10: 561–591 1590996 10.1146/annurev.iy.10.040192.003021

[bib14] Ponta H, Sherman L, Herrlich PA (2003) CD44: from adhesion molecules to signalling regulators. Nat Rev Mol Cell Biol 4: 33–45 12511867 10.1038/nrm1004

[bib15] Rouzier R, Perou CM, Symmans WF, Ibrahim N, Cristofanilli M, Anderson K, Hess KR, Stec J, Ayers M, Wagner P, Morandi P, Fan C, Rabiul I, Ross JS, Hortobagyi GN, Pusztai L (2005) Breast cancer molecular subtypes respond differently to preoperative chemotherapy. Clin Cancer Res 11: 5678–5685 16115903 10.1158/1078-0432.CCR-04-2421

[bib16] Shen WH, Zhou JH, Broussard SR, Freund GG, Dantzer R, Kelley KW (2002) Proinflammatory cytokines block growth of breast cancer cells by impairing signals from a growth factor receptor. Cancer Res 62: 4746–4756 12183434

[bib17] Subramaniam V, Vincent IR, Gilakjan M, Jothy S (2007) Suppression of human colon cancer tumors in nude mice by siRNA CD44 gene therapy. Exp Mol Pathol 83: 332–340 17945212 10.1016/j.yexmp.2007.08.013

[bib18] Tang B, Yoo N, Vu M, Mamura M, Nam JS, Ooshima A, Du Z, Desprez PY, Anver MR, Michalowska AM, Shih J, Parks WT, Wakefield LM (2007) Transforming growth factor-beta can suppress tumorigenesis through effects on the putative cancer stem or early progenitor cell and committed progeny in a breast cancer xenograft model. Cancer Res 67: 8643–8652 17875704 10.1158/0008-5472.CAN-07-0982 PMC2427144

[bib19] Underhill-Day N, Heath JK (2006) Oncostatin M (OSM) cytostasis of breast tumor cells: characterization of an OSM receptor beta-specific kernel. Cancer Res 66: 10891–10901 17108126 10.1158/0008-5472.CAN-06-1766

